# Instant improvement in monaural spatial hearing abilities through cognitive feedback

**DOI:** 10.1007/s00221-022-06333-7

**Published:** 2022-03-03

**Authors:** Tine Arras, Hillary Snapp, Anouk Sangen, Chantal Snels, Iris Kuntz, Tinne Theunen, Kiana Kheirkhah, Andrzej Zarowski, Thomas Wesarg, Astrid van Wieringen, Martijn J. H. Agterberg

**Affiliations:** 1 Department of Neurosciences, Experimental ORL, KU Leuven, University of Leuven, Leuven, Belgium; 2 Department of Otolaryngology, University of Miami, Miami, FL USA; 3 Department of Otorhinolaryngology, Donders Institute for Brain, Cognition and Behaviour, Radboudumc, Nijmegen, The Netherlands; 4 Department of Otorhinolaryngology, University Ghent, Ghent, Belgium; 5 Department of Otorhinolaryngology—Head and Neck Surgery, Medical Center—University of Freiburg, Faculty of Medicine, University of Freiburg, Freiburg im Breisgau, Germany; 6 ENT Department Sint-Augustinus Antwerp, European Institute For ORL, Antwerp, Belgium; 7 Department of Biophysics, Donders Institute for Brain, Cognition and Behaviour, Radboud University, Heyendaalseweg 135, 6525 AJ Nijmegen, The Netherlands

**Keywords:** Directional hearing, Monaural, Sound level, Timbre, Top-down information

## Abstract

Several studies report that sound localization performance of acute and chronic monauralized normal-hearing listeners can improve through training. Typically, training sessions are administered daily for several days or weeks. While this intensive training is effective, it may also be that monaural localization abilities improve instantly after providing explicit top-down information about the direction dependent change in timbre and level. The aim of the present study was to investigate whether cognitive feedback (i.e., top-down information) could instantly improve sound localization in naive acutely monauralized listeners. Forty-three normal-hearing listeners (experimental group), divided over five different centers, were tested. Two control groups, consisting of, respectively, nine and eleven normal-hearing listeners, were tested in one center. Broadband sounds (0.5–20 kHz) were presented from visible loudspeakers, positioned in azimuth (− 90° to 90°). Participants in the experimental group received explicit information about the noticeable difference in timbre and the poor localization in the monauralized listening condition, resulting in an instant improvement in sound localization abilities. With subsequent roving of stimulus level (20 dB), sound localization performance deteriorated immediately. The reported improvement is related to the context of the localization test. The results provide important implications for studies investigating sound localization in a clinical setting, especially during closed-set testing, and indicate the importance of top-down information.

## Introduction

**Fig. 1:**
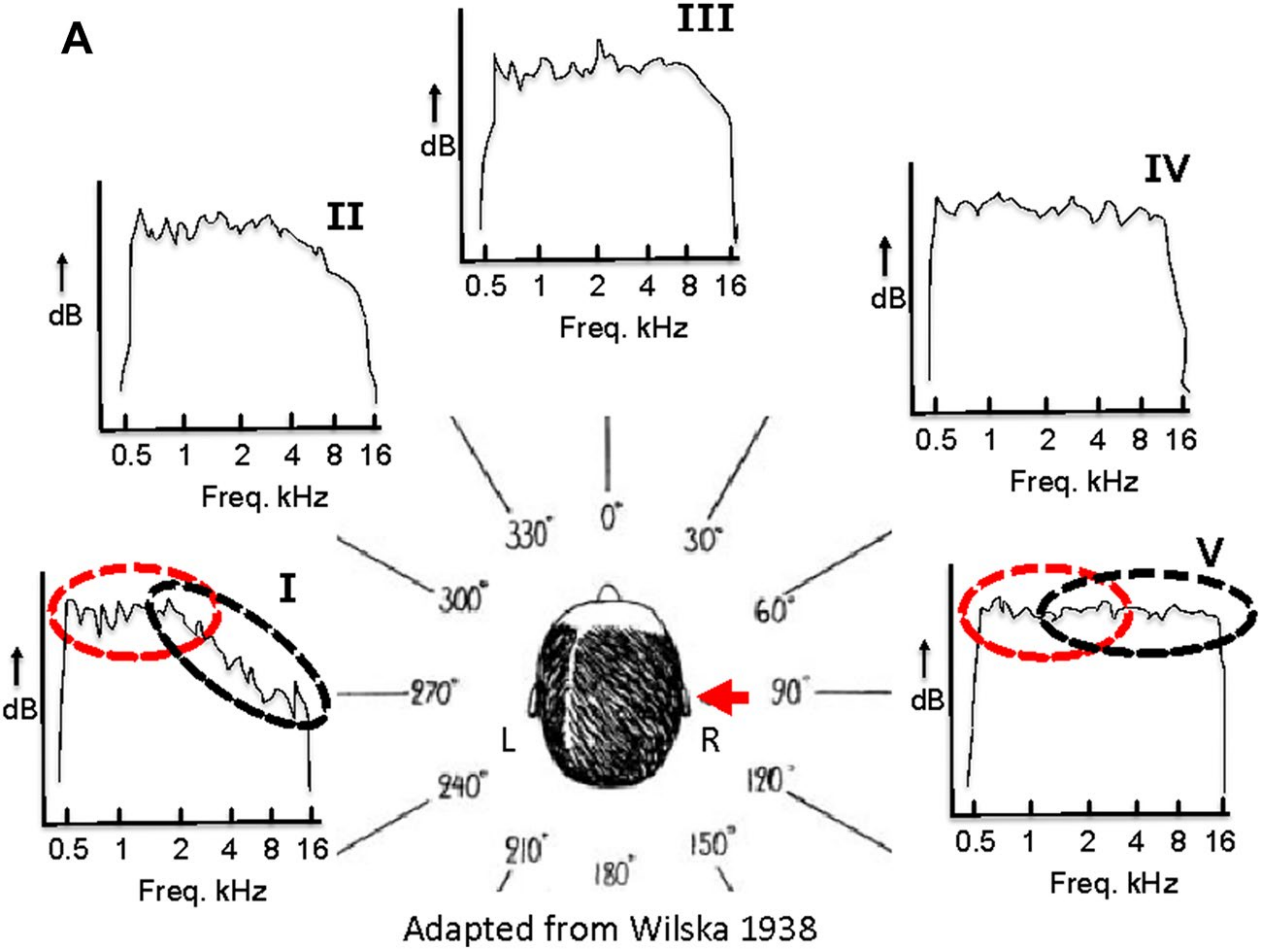
Schematic illustration (adapted from Wilska 1938) indicating the low-pass filtering by the head (subfigure I, red dotted circle). The full spectrum of the signal is perceived at the right ear directed to the sound source (red arrow, subfigure V), while the high frequency components are increasingly attenuated as the positions in azimuth are more distal to the source (subfigures II, III and IV), inducing a change in timbre

The current study focuses on an underestimated monaural cue (i.e., timbre) arising from the head shadow. When a broadband (BB) sound is presented to the hearing-impaired side of a monauralized listener, the frequency-dependent attenuation provides a physical cue due to the low pass filtering by the head, characterized by a change in timbre (Fig. 1). This change in timbre is noticeable when fixed flat-spectrum stimuli are presented (Stevens and Newman 1936; Wilska 1938; Wightman and Kistler 1997; Shub et al. 2008).

While many studies address the role of pinna-related spectral cues (e.g., Shub et al. 2008), the noticeable difference in timbre in a monaural listening condition is often overlooked. Timbre can be an important cue and explicit information about the direction dependent change in timbre can have an immediate effect on the monaural listeners’ ability to localize sounds. This monaural cue probably plays an important role in clinical studies in which an improvement in sound localization is realized by training (Luntz et al. 2005; Firszt et al. 2015; Bonne et al. 2019).

In the acute monaural hearing condition, binaural processing of interaural time differences (ITDs) and interaural level differences (ILDs) is heavily distorted and listeners perceive the stimuli mainly at the hearing (unplugged) side (Angell and Fite 1901; Musicant and Butler 1984; Oldfield and Parker 1986; Slattery and Middlebrooks 1994; Blauert 1997; Wightman and Kistler 1997; Gordon and Kral 2019). Training can improve sound localization abilities in this acute monaural hearing condition. Typically, several days of training with a few hundred training trials per day were needed to demonstrate an improvement in azimuth localization (Musicant and Butler 1980; Kumpik et al. 2010; Irving and Moore 2011; Strelnikov et al. 2011; Keating et al. 2016; Zonooz and Van Opstal 2019; Rabini et al. 2019; Valzolgher et al. 2020). Notably, during most of these studies participants wore their ear plug only during testing (see Table 1 for study characteristics). Two studies investigated the effect of training in listeners with a chronic unilateral earplug (Kumpik et al. 2010; Irving and Moore 2011).

**Table 1:** Characteristics of eight studies investigating the effect of training on free field sound localization of unilaterally plugged normal-hearing listeners

	Earplug	Number of subjects	Training days	Stimuli	Roving	Loudspeaker positions	Range	Feedback
Musicant and Butler (1980)	During sessions	8	10 days	Train HP pulses	None	15 degrees	90°	No feedback
Kumpik et al. (2010)	Chronic	20	7–8 days	Gaussian noise (0–20 kHz), 300 ms	50, 56, 63, 70, 77, 84 dB	30 degrees	360°	Colored marker
Irving and Moore (2011)	Chronic	12	8 days	Pink noise, 40, 100 and 500 ms	50–70 dB	15 degrees	360°	Flashing screen
Strelnikov et al. (2011)	During sessions	18	5 days	White noise 50 ms	60 dB SPL	10 degrees	140°	Visual “correct” “incorrect”
Keating et al. (2015)	During sessions	11	7 sessions within 3 weeks	Broadband noise, 100 ms	49–77 dB SPL	30 degrees	360°	Green or Red flash
Rabini et al. (2019)	During sessions 1,3 and 5	45	5 days	Italian syllable, 500 ms	None	3 degrees	43°	Visual
Zonooz and Opstal (2019)	During sessions	8	> 3 days	High-pass	50, 60, 70 dB	15 degrees	120°	Green LED
Valzolgher et al. (2020)	During session	16	3 days	White-noise, 500 ms	62 dB	7.2 degrees	120°	Kinesthetic

In general, during training days, feedback from other senses was provided in the form of flashing lights (i.e., visual feedback). These lights indicated the position of the correct loudspeaker or whether a response was correct or incorrect. Several studies demonstrated that feedback from other modalities than the visual system could further improve the monaural localization abilities. For example, kinesthetic cues improve monaural localization abilities when subjects pay attention to the position of sounds (i.e., active training), and these multisensory cues are beneficial for acute monaural listeners (Valzolgher et al. 2020).

There is little agreement on the type of cues, duration of training and method of training needed to improve localization abilities in unilaterally plugged normal-hearing listeners. In clinical setups and in setups in which subjects are trained to improve their localization abilities, loudspeakers are visible and/or the position of loudspeakers is indicated (i.e., closed-set testing). This might result in confounding situations, because participants might perceive sounds from directions that do not correspond with a loudspeaker position. The human neural system is continuously updating available information and unilateral plugged listeners can learn to use monaural cues and pinna-related spectral cues to optimize azimuthal localization in a monaural hearing condition (Wright and Zhang 2006; Kumpik et al. 2010; Keating and King 2015).

In the present study, explicit top-down information, in the form of cognitive feedback, is provided to acutely monauralized listeners to increase the participants’ knowledge about the acute monaural listening condition. We hypothesize that this cognitive feedback can instantly improve sound localization abilities in an acute monaural listening condition as this type of feedback assists the learner to reflect on their learning strategies.

Normal-hearing participants (*n* = 43) received a unilateral ear plug and horizontal sound localization was evaluated in four conditions in a within-subject experimental design. Sound localization was tested in the normal-hearing condition (condition 1), in the acute monaural hearing condition before and after receiving explicit information regarding the monaural hearing condition (conditions 2 and 3, respectively), and finally in a condition in which stimuli were presented at three different sound levels (condition 4).

The top-down information consisted of a brief explanation of the monauralized hearing condition and the available monaural cues, combined with a short exposure to the stimuli. Participants were told that all stimuli would be perceived at the side of the open ear, while stimuli were actually presented from all loudspeaker locations. The change in timbre of the stimulus was explained in layman’s terms, and participants were exposed to 15 broadband stimuli originating from the five visible loudspeaker positions. Results were compared with two control groups not receiving cognitive feedback.

For clarity, with the terms “cognitive feedback” and “explicit top-down information” we refer to providing contextual information about the acute monaural hearing situation to the participants. The contextual information consisted of two parts. (i) It was explained that they could perceive a change in timbre. (ii) It was told that their performance was poor and that they localized most of the sounds toward the unplugged ear, while stimuli were presented from all loudspeaker locations (i.e., feedback on their poor performance).

## Materials and methods

### Participants

Sixty-two adult participants (about 50% female, aged 21–57 years) were included in the study. None of the participants reported a history of inferior hearing or neurological disease, all had normal or corrected to normal vision. Forty-three were tested while providing explicit information (i.e., cognitive feedback), nine were tested in a control condition without theoretical or practical orientation to the task, and eleven were tested without orientation to the task but with exposure to the 15 stimuli presented between condition 2 and 3. The experiment was conducted at five centers to determine the robustness of the effects, replicability and potential generalization to other sites performing localization assessments. All participants receiving cognitive feedback (Clinic A, *n* = 10; Clinic B, *n* = 10; Clinic C, *n* = 10; Clinic D, *n* = 6, Clinic E, *n* = 7) and all participants in the control groups (Clinic B, *n* = 20) were naive to the experimental conditions and had normal-hearing bilaterally as determined by air-conduction hearing thresholds < 20 dB HL across the standard audiometric test frequencies, 250–8000 Hz. All experimental protocols adhered to the guidelines of the universities’ local ethics committees.

### Control condition

As a control for the hypothesis two control experiments were performed. Nine normal-hearing participants underwent localization testing under the same experimental conditions as the experimental group, without receiving any top-down information on the monaural hearing condition and without exposure to the 15 BB stimuli between condition 2 and 3 (see Sect. 2.5). In a second control group, eleven normal-hearing participants were tested without receiving cognitive feedback but with exposure to the 15 stimuli. These experiments were conducted at the Experimental ORL, under the same test conditions as in ten participants of the experimental group, also assessed at this center.

### Test setups

**Fig. 2:**
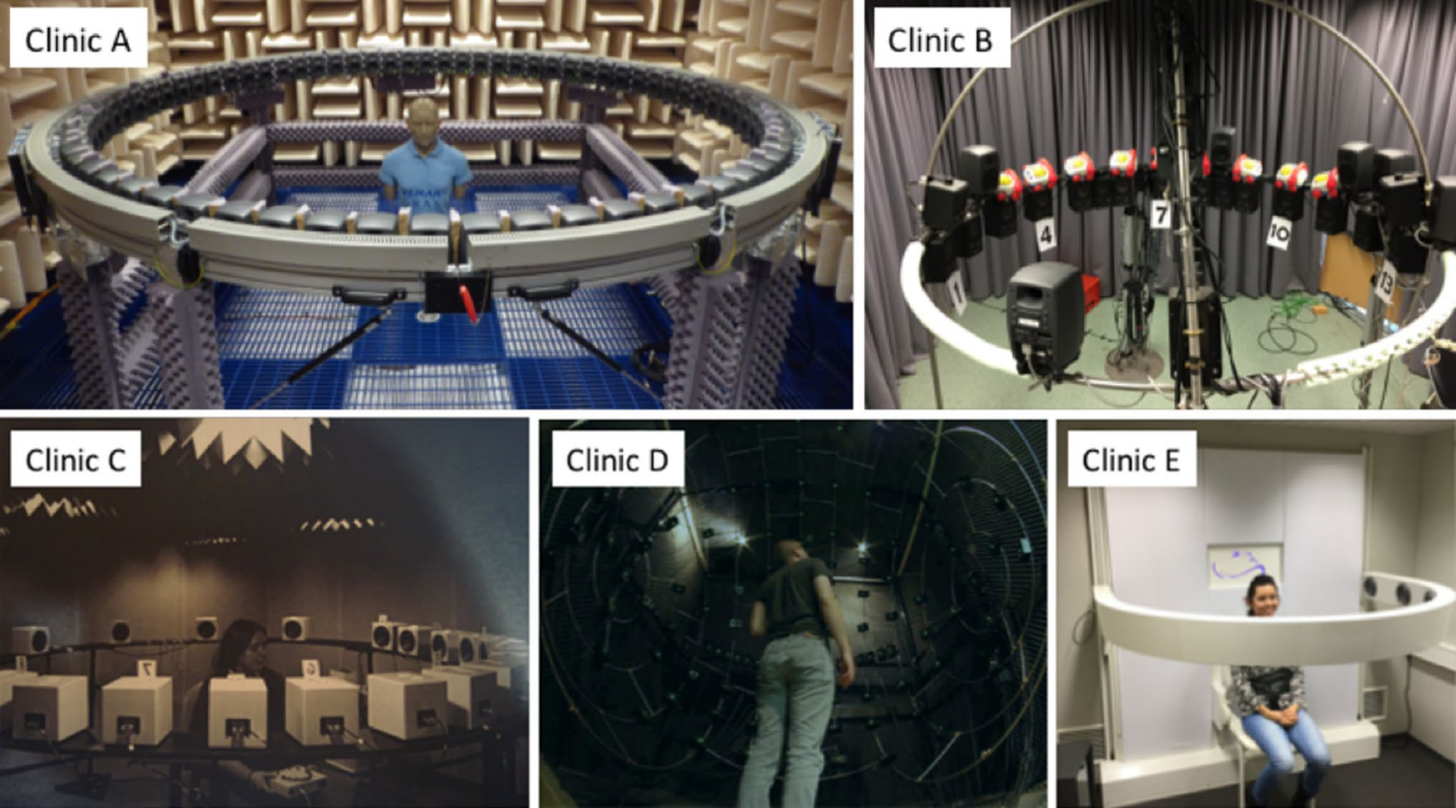
Illustrations of the different test setups used in the five clinics. All sites used a horizontal array, and at each site the loudspeakers were clearly indicated with a visual marker. At each site, the distance between the loudspeaker and the participant was 1 m at minimum

The test setups are depicted in Fig. 2. At each site sound localization was tested in a closed-set paradigm in which the loudspeakers were clearly indicated with visual markers. In center C, participants used an indicator box to indicate the loudspeaker number. In centers A, B, D, and E, participants were asked to verbally identify the loudspeaker number. Participants were instructed to maintain their head in a forward position, facing a 0° azimuth symbol or LED, prior to stimulus presentation, and asked to “head point” to the perceived sound location and indicate the number of the loudspeaker after each stimulus was presented. All sites used a horizontal array with stimuli presented in the frontal hemifield spanning ± 90°. A minimum distance of 1 m between the loudspeakers’ front and the center of the participants’ head was maintained.

### Stimuli and experimental procedure

**Fig. 3:**
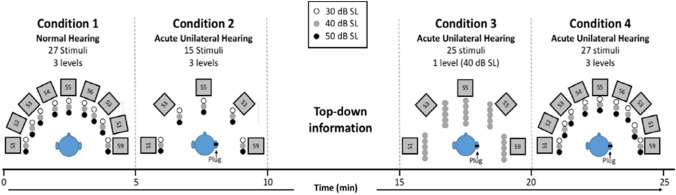
Schematic representation of the experimental procedure. All stimuli presented in the total experiment are indicated as single dots. Stimulus sound level is indicated with black, gray and white dots. In conditions 2, 3 and 4, participants are unilaterally plugged. The experimental group received cognitive feedback (top-down information in about 5 min) immediately prior to condition 3. For the sake of clarity, the small difference in some loudspeaker locations (2.5°) between clinic A and the other clinics, is not indicated. Time scale indicated. *SL* sensory level

All five centers used the same stimulus in all experimental conditions; a 0.5–20 kHz BB Gaussian white noise, custom generated using MATLAB, Version 7.4, The Mathworks, Natick, MA, USA, and saved as WAV file. Stimuli were randomly presented in azimuth. Possible levels were 30, 40 and 50 dB SL. Prior to testing, SL was determined by plugging and muffing both ears and presenting the BB stimulus from a location in front of the participant. Stimuli were 150 ms in duration. Note that because listeners were asked to fix their heads at the center loudspeaker and 150-ms stimuli were presented, it was ensured the participant’s head remained stationary during stimulus presentation (Wasmann et al 2020). A total of 94 stimuli were presented during the four conditions. Participants always started with the normal-hearing condition, followed by three acute unilateral-plug conditions (see Fig. 3). Half of participants were plugged to the right and the other half to the left. An experimental session lasted approximately 25 min.

### Conditions and cognitive feedback

Localization testing was conducted in a normal-hearing condition (condition 1) and three acute unilateral-plug conditions (conditions 2–4). In the plug conditions one ear was plugged and muffed (simulating acute unilateral hearing loss). Stimuli characteristics, including number and level of stimuli is shown in Fig. 3, along with the source location of the stimuli for each condition.

In condition 1, stimuli were randomly presented at 30, 40 or 50 dB SL from each of nine loudspeaker locations, totaling 27 stimuli (Fig. 3).

**Fig. 4:**
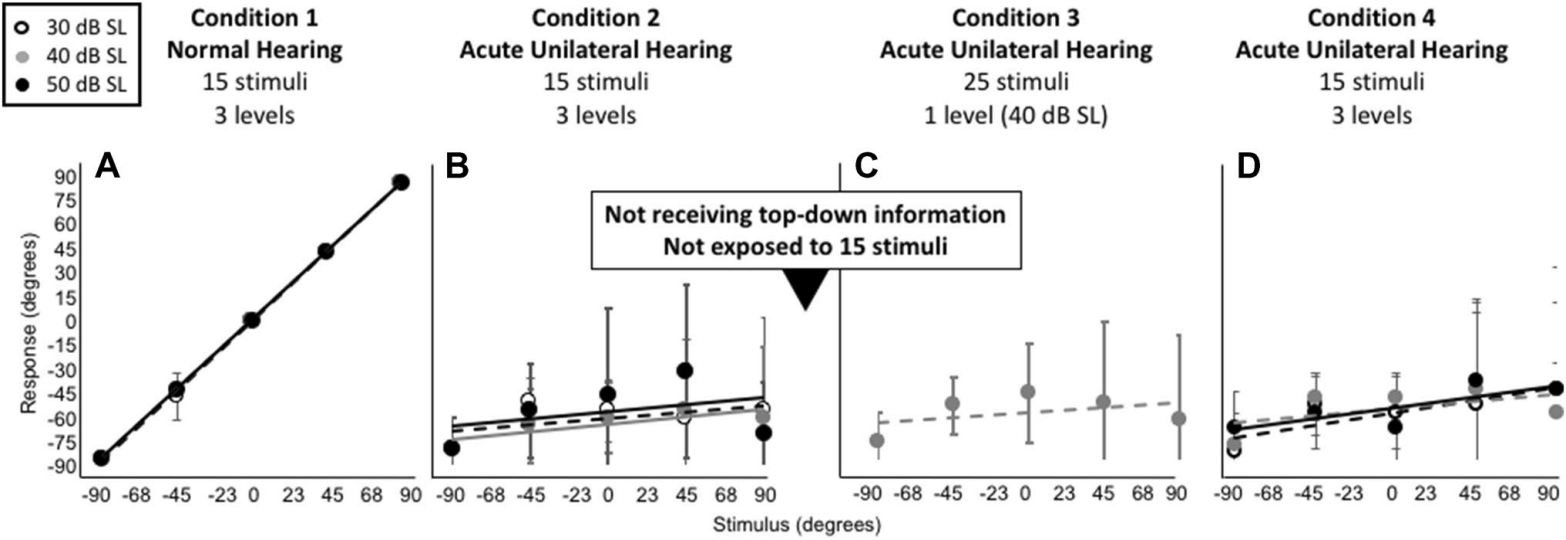
Average stimulus–response plots (± standard deviation) for nine control listeners who did not receive cognitive feedback (i.e., who did not receive top-down information). Note the remaining clear leftward bias (**B**–**D**)

**Fig. 5:**
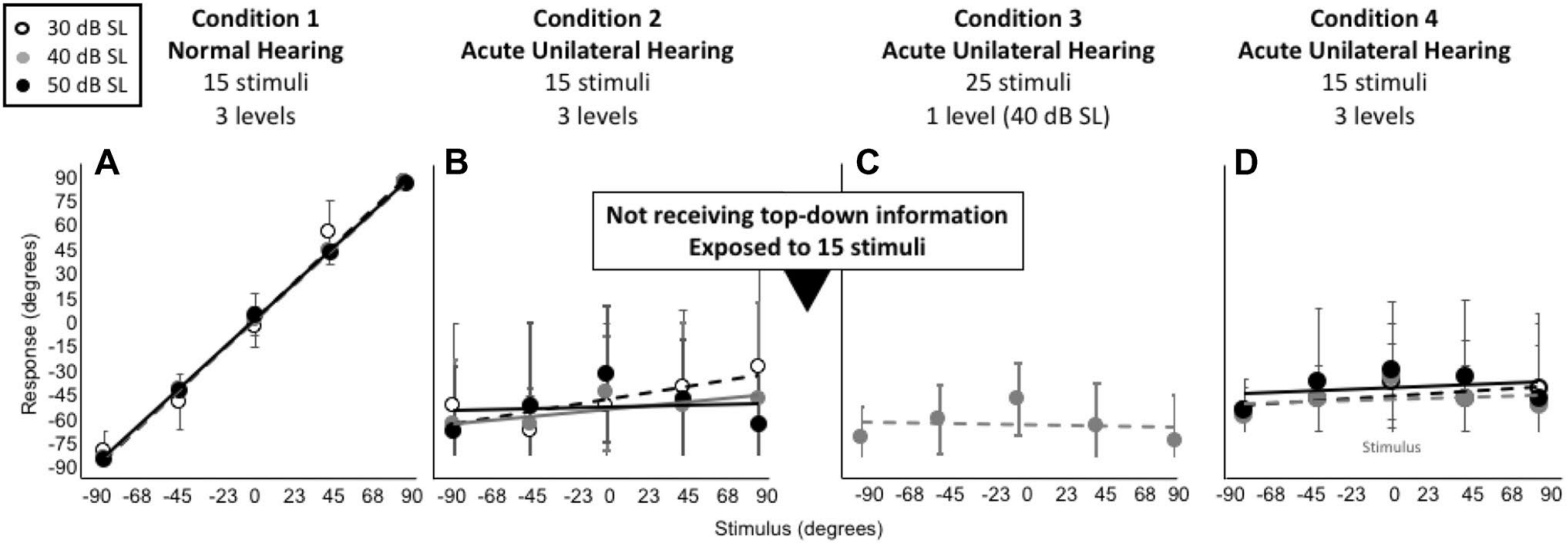
Average stimulus–response plots (± standard deviation) for eleven control listeners who were exposed to 15 stimuli between condition 2 and 3 but who did not receive cognitive feedback (i.e., who did not receive top-down information). Note the remaining clear leftward bias (**B**–**D**)

**Fig. 6:**
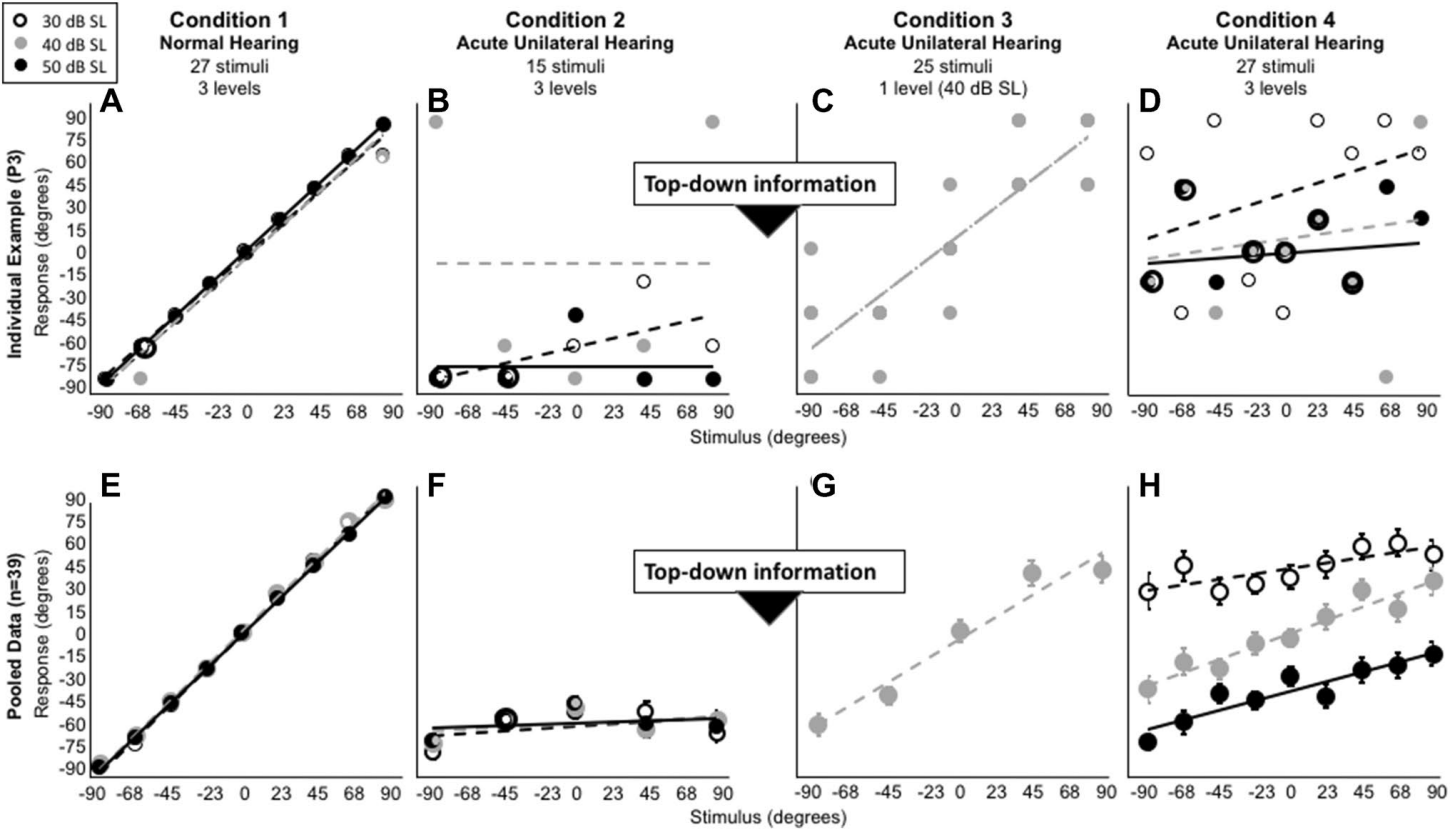
Individual stimulus–response plots (**A**–**D**) and the average stimulus–response plots (± standard deviation) in all four conditions for all 39 participants (**E**–**H**). Note the clear leftward bias (**B**, **F**) prior to providing the top-down information, and the improved sound localization after providing top-down information (**C**, **G**). When stimuli were presented at three levels (**D**, **H**) sound localization deteriorated immediately. Particularly at group level, a clear level-dependent bias is visible (**H**)

In condition 2 stimuli were again randomly presented at 30, 40 or 50 dB SL, but from only five loudspeakers, totaling 15 stimuli (Figs. 4B, 5B and 6B, F). Testing began immediately after plugging one of the ears.

Following condition 2 and immediately prior to condition 3, the experimental group received top-down information (cognitive feedback) regarding the monaural localization task. Participants were provided with information about their (inaccurate) localization performance demonstrated during the acute unilateral hearing condition (condition 2). Specifically, it was explained that they localized the stimuli, as predicted, mainly towards the side of their normal-hearing (open) ear. Using layman’s terms, participants were then informed about the acoustic head-shadow. It was explained that sounds would be perceived different in timbre when originating from different locations. The head acts as a low-pass filter resulting in head-related frequency dependent damping of stimuli originating from the plugged side. Therefore, broadband noise is more perceived like *psss* when presented from the hearing side and more like *pshh* when presented at the side of the plugged ear. Note that this direction-specific subtle change in timbre was already described by Stevens and Newman in 1936. To ensure that the participants understood the provided information, they were exposed to a maximum of 15 stimuli, presented at 40 dB SL, prior to test condition 3. These stimuli were presented in sequential order from five clearly indicated loudspeaker positions (90°, 45°, 0°, − 45°, and − 90°), allowing the subject to perceive the change in loudness and timbre as the signal moved from one side of the head to the other. Altogether, providing participants with information and exposure to the 15 stimuli lasted approximately 5 min.

In condition 3 participants were asked to localize stimuli presented at 40 dB SL only. Sounds were presented at 40 dB only, because a change in timbre is better detectable when sound level is not roved. Twenty-five stimuli were presented at random from five loudspeaker locations (five stimuli from each loudspeaker).

In condition 4, the experimental procedure of condition 3 was repeated without informing the participants that stimuli were randomly presented at 3 levels (30, 40 and 50 dB SL), and that the number of loudspeaker locations was increased from five back to nine (see Fig. 2). Participants received verbal encouragements without providing any specific information regarding their performance.

### Data analysis

The individual data (*n* = 63) were checked for consistency and adherence to the protocol. In total, four protocol deviations were identified, resulting in a total of 39 data sets in the experimental group and 20 data sets in the control groups. Individual data were adjusted so that responses corresponded to right-ear plugging and then grouped for analysis. The mean absolute errors of the stimulus–response relations were calculated and analysis of variance (ANOVA) was used to assess the effects.

## Results

Figures 4 and 5 show the pooled stimulus–response relationships for the control participants (*n* = 9; Fig. 4 and *n* = 11; Fig. 5) for each condition. As expected, target response accuracy was high for condition 1 (Figs. 4A, 5A). Following acute plugging, the control groups demonstrate a strong lateralization towards the open ear for all monauralized listening conditions (Figs. 4B–D, 5B–D). Exposure during condition 3 (Fig. 4C) and 4 (Fig. 4D) did not result in a significant change in localization behavior, demonstrating a continued reliance on highly disturbed binaural cues. Exposure to 15 stimuli between condition 2 and 3 did not affect localization performance (compare Fig. 4C, D with Fig. 5C, D).

Figure 6 shows results of the experimental group (*n* = 39) for each condition. Here, an individual participant example (upper row), and the pooled stimulus–response relationship for all 39 participants (lower row) is presented. The stimulus–response relationship for the normal-hearing condition (Fig. 6A, E) is consistent with that observed in the control groups (Figs. 4A and 5A), and demonstrates the typical diagonal orientation of the regression line indicating accurate localization. ANOVA on mean absolute error (MAE) for the pooled data yielded a significant main effect of condition, *F*(3, 152) = 136.98, *p* < 0.0001. Post hoc comparisons (Table 2) using the Tukey Honest Significant Difference (HSD) test indicated that the MAE under normal-hearing conditions (3.1°) was significantly lower (better) than under any of the simulated hearing loss conditions (64°, *p* < 0.0001). Normal-hearing localization abilities were observed across all levels as demonstrated by a high degree of stimulus–response accuracy.

**Table 2:** Multiple comparisons between test conditions

Condition 1	Condition 2	Mean Diff	Std Err Diff	Lower CL	Upper CL	*p* value
C1 NH	C2 Acute UH	61.08	3.2	52.78	69.39	< .0001
	C3 Acute UH	28.35	3.2	20.04	36.67	< .0001
	C4 Acute UH	47.55	3.2	39.24	55.86	< .0001
C2 Acute UH	C3 Acute UH	32.73	3.2	24.42	41.04	< .0001
	C4 Acute UH	13.53	3.2	5.22	21.85	< .0002
C3 Acute UH	C4 Acute UH	19.2	3.2	10.88	27.51	< .0001

One-way ANOVA on MAE for the pooled data yielded significant variation among conditions, *F*(3, 152) = 136.98, *p* < 0.0001. Post hoc comparisons using the Tukey HSD test is presented

*UH* unilateral hearing

In the acute unilateral-plug condition (Fig. 6B, F), a strong open-ear bias was observed, reflected by the negative response azimuth values between − 45° and − 90° (Fig. 6F, MAE = 64.2°). Localization in this acute unilateral-plug condition was significantly worse than for normal hearing (Tukey HSD test, *p* < 0.0001). At the individual level it is visible that stimuli were not always perceived toward the hearing ear (Fig. 6B).

In condition 3 (Fig. 6C, G) the overall MAE (31°) was considerably smaller than for condition 2 (64°, *p* < 0.0001). Unlike that observed in the controls, a clear improvement and highly accurate localization performance is indicated by the diagonal orientation of the data points (Fig. 6G). The control groups continued to demonstrate a strong response bias toward the open ear (Figs. 4C, 5C).

Figure 6D, H (condition 4) demonstrates the disruption in localization accuracy that occurred with roving of sound level. Review of grouped data (Fig. 6H) shows that stimuli presented at 30 dB SL were perceived towards the plugged side, and stimuli presented at 50 dB SL towards the side of the unplugged ear. Interestingly, participants were more accurate in localizing stimuli presented at 40 dB SL (diagonal orientation of the data), though performance does not reach that which is observed in condition 3 (i.e., comparison grey regression line Fig. 6G with 6H). The data demonstrates that, when the stimuli are roved over a 20-dB range, the monaural cue becomes unreliable.

**Fig. 7:**
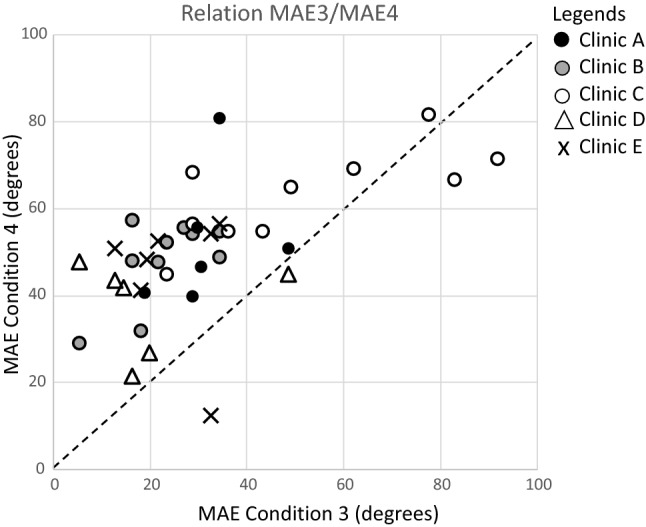
Relation between MAE obtained in condition 3 and in condition 4 in all 39 participants. Most data points lie above the diagonal, indicating better localization abilities in condition 3 compared to condition 4

Figure 7 shows that the experimental group had smaller (better) MAEs in condition 3 than in condition 4 (most points lie above the diagonal), demonstrating that overall sound localization was better in condition 3 compared to condition 4. Furthermore, the figure reveals that the whole data set contains only a few outliers, tested in clinic C, with an MAE > 60°.

## Discussion

### Instant improvement in sound localization

The present study shows that providing participants (*n* = 39) with explicit top-down information about the acute monaural hearing situation, in combination with minimal exposure to 15 BB noise bursts, instantly improved monaural localization abilities. Introducing roving of sound level re-disrupted monaural localization (Fig. 6H). Nine and eleven participants, tested under the same experimental conditions without receiving cognitive feedback (Fig. 4), and with exposure to 15 stimuli (Fig. 5), did not demonstrate any improvement in localization.

### Training

The eight studies listed in Table 1 demonstrated improved monaural localization abilities after training (Musicant and Butler 1980; Kumpik et al. 2010; Irving and Moore 2011; Strelnikov et al. 2011; Keating et al. 2016; Zonooz and Van Opstal 2019; Rabini et al. 2019; Valzolgher et al. 2020). These studies showed a practice effect and the reweighting of monaural spectral information. Kumpik et al. (2010) demonstrated that, after daily training sessions with visual feedback for at least 1 week, monauralized normal-hearing listeners acquire the ability to use monaural spectral cues for the localization of sounds in azimuth. Furthermore, they demonstrated that there was no clear evidence for adaptation to altered ITDs and ILDs. Interestingly, recent studies demonstrated that simple test–retesting, without providing the visual feedback, resulted in an improvement in monaural localization abilities (Rabini et al. 2019; Zonooz and Van Opstal 2019).

Typically, in all the studies mentioned above, participants were exposed to a large number of stimuli during the training and testing sessions (> 1300 stimuli). Only Musicant and Butler (1980) adopted a procedure in which participants received as limited as possible additional auditory experience in their setup. Still, participants were exposed to 660 stimuli. Note that in the present study participants were exposed to only 109 stimuli (duration experiment about 25 min).

Recently, several studies showed that sensory feedback from other modalities than the visual system could further improve sound localization (Fletcher et al. 2020; Valzolgher et al. 2020). For example, a striking improvement in monaural localization is demonstrated in participants who moved an audio-bracelet, attached to their wrist, while paying attention to the direction-dependent sounds emitted by this audio bracelet (Valzolgher et al. 2020).

**Fig. 8:**
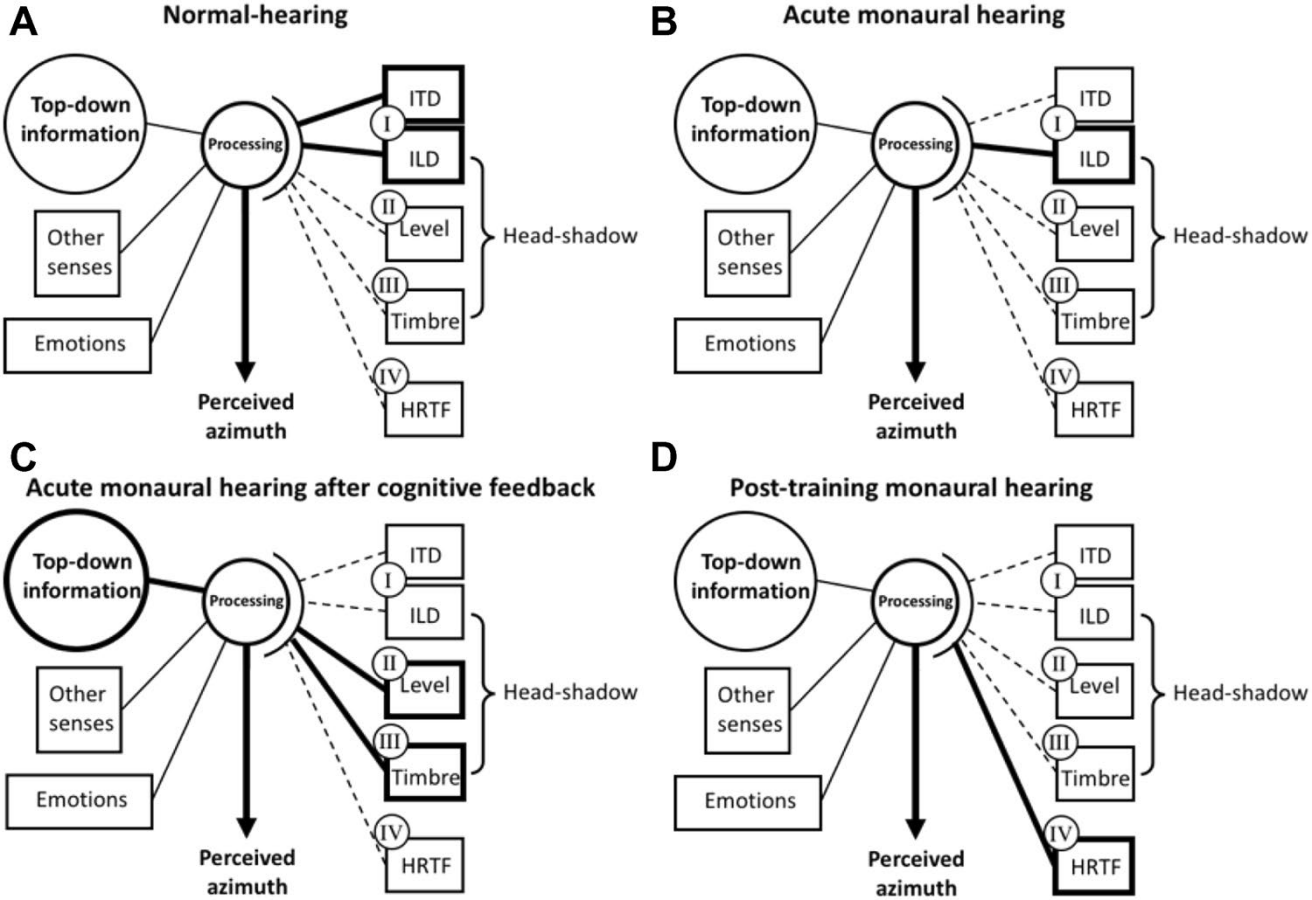
Schematic drawing of a general model indicating the dominant mechanisms underlying the performance in the normal-hearing condition (**A**), acute monaural hearing condition (**B**), acute monaural hearing condition after cognitive feedback (**C**) and the post-training monaural hearing condition (**D**). Potentially, four acoustic cues (I, II, III and IV) can contribute to localization in azimuth. In the monauralized conditions several cues and or factors can become dominant. *ITD* Interaural time differences, *ILD* Interaural level differences, *HRTF* head-related transfer functions

These above-mentioned studies differ in many aspects from each other (see Table 1), and it remains unclear which (monaural) cues are dominant. A general model, adapted from the conceptual model (Blauert 1997; Hofman and Van Opstal 1998; Zonooz and Van Opstal 2019), indicates how top-down information and information from other senses can heavily affect the processing of altered binaural cues (Fig. 8). The model extends previous models describing factors within the auditory system (Hartmann et al. 1998a; Braasch 2016) by adding the contribution of timbre. In the normal-hearing condition (Fig. 8A) sound localization is optimal because of the accurate processing of binaural cues (i.e., ITDs and ILDs). This is indicated by the bold solid lines (I) and is reflected by the accurate localization in condition 1 (Figs. 4A, 5A and 6E). In the acute monaural hearing condition (Fig. 8B), the ITDs and ILDs are highly disturbed, indicated by the ‘bold dashed’ and ‘bold solid’ line (I), resulting in a strong bias towards the hearing ear because of an extreme ILD (see condition 2, Figs. 4B, 5B and 6F). The present study demonstrates the instant improvement in condition 3 (Fig. 6G) based on the use of timbre and level after providing cognitive feedback (bold solid lines, Fig. 8C). Training with visual and/or haptic feedback (Irving and Moore 2011; Strelnikov et al. 2011; Oldegaard et al. 2015; Fletcher et al. 2020; Valzolgher et al. 2020) can change the behavior resulting in reasonable localization abilities when stimuli are presented at one single level, and several studies indicated that monauralized listeners can learn to use spectral pinna cues (i.e., HRTFs) for localization of sounds in azimuth (Kumpik et al. 2010; Keating et al. 2016) indicated by the ‘bold solid’ line in Fig. 8D. Note that this post-training effect is not part of the current study.

The presented model displays the multidisciplinary aspects of the presented work. The contribution of top-down processing in spatial hearing is important, although not yet fully understood (Souffi et al. 2021). Studying “cognitive feedback” in this field of research is complex and there is still a lot to gain, since deep knowledge in psychology, metacognition (Flavell 1979; Dunlosky and Hertzog 2000), neuroscience and audiology is needed to really understand which factors are crucial in training sound localization abilities. For example, when participants are unaware that the conditions are changed while being tested, knowledge updating cannot occur. Monitoring their accuracy during the test, metacognitive experience and the ability to test assumptions can all have significant effects on their localization performance.

### Possible mechanisms underlying monaural localization abilities

In many clinical studies, and in studies with unilaterally plugged normal-hearing listeners, it remains uncertain which of the possible (auditory) cues are key in the reported improvements. Especially in plugged normal-hearing listeners (Wightman and Kistler 1997) but also in patients with unilateral aural atresia (Agterberg et al. 2012; Kumpik and King 2019; Thompson et al. 2020; Canfarotta et al. 2021) remnant binaural cues can explain accurate localization of high-level sounds. Apparently, remnant binaural cues are not important in the presented acute monaural condition, since all stimuli are perceived extremely towards the open ear (Figs. 4B, 5B, F). In the present study, unbeknownst to the listener, during the last test condition the signal was roved over three levels spanning a 20-dB range (Fig. 3). Compared to the test condition in which the stimuli were not roved, overall localization deteriorated, with the overall MAE increasing from 31° in condition 3 to 51° in condition 4 (*p* < 0.0001). However, localization for the 40 dB stimuli rarely differed between both conditions. The results demonstrate that when the level is roved, using sound level becomes an inaccurate strategy, resulting in a bias towards the open normal-hearing ear for high-level stimuli and a bias towards the plugged ear for low-level stimuli (Fig. 6H). In contrast, this effect of sound level was not present for the control groups who did not receive any top-down information regarding the monauralized listening condition (Figs. 4D, 5D).

The demonstrated instant improvement is related to the context of the localization test. Top-down information regarding the sound locations and knowing that sounds are presented at one sound level provided useful information that can be used instantly. When the levels are roved sound localization deteriorates. In this experiment this deterioration is related to the fact that the participants were not aware that we changed the experimental condition. In other words, the reported improvement is related to the context of the experiment and does not necessarily reflect an improvement in localization skills, and more research is needed to investigate generalization of results obtained in laboratory settings to real-world outcomes (Risoud et al. 2019).

### Factors affecting sound localization

When investigating sound localization abilities, the sound level is often not roved (Bosman et al. 2001; Luntz et al. 2005; Kitterick et al. 2011; Hansen et al. 2013; Kuhnle et al. 2013; Litovsky et al. 2013; Monini et al. 2015; Parisa et al. 2017; Asp et al. 2018; Eklof et al. 2018; Yang et al. 2018; Bonne et al. 2019; Zirn et al. 2019; Valzolgher et al. 2020), or only roved over a small (< 10 dB) range (Van Deun et al. 2010; Murphy et al. 2011; Nawaz et al. 2014; Firszt et al. 2015; Snapp et al. 2017; Gawliczek et al. 2018; Fletcher et al. 2020). In the absence of sufficient roving, listeners can learn to use the overall level as a cue (Middlebrooks and Green 1991). Moreover, with insufficient roving (i.e., roving < 20 dB) of broadband stimuli a salient change in timbre can be used as cue (Wightman and Kistler 1997; Shub et al. 2008). It would be of interest to test the localization ability of acute monauralized normal-hearing listeners with an additional mold in the pinna of the hearing ear. It is expected that in this condition the participants would still demonstrate the instant improvement in localization abilities. In contrast, when participants would suffer a high frequency hearing loss an improvement is not expected, because listeners with presbycusis do not perceive the change in timbre.

To what extent factors such as signal bandwidth (Butler 1986), stimulus level (Macpherson and Middlebrooks 2000; Sabin et al. 2005), visibility of loudspeakers, response method (Populin et al. 2008; Bahu et al. 2016), subjective certainty (Rabini et al. 2020), head movements (Pastore et al. 2020), age (Freigang et al. 2014), sensory (Oldegaard et al. 2015) and motor related input (Valzolgher et al. 2020), pinna cues (Batteau 1967; Shub et al. 2009), reflecting surfaces (Hartmann et al. 1998), experimental design, and top-down information contribute to acute and chronic monaural sound localization abilities requires further study. Furthermore, it would be of interest to study the different aspects of the top-down and bottom-up information in more detail.

Regarding the influence of vision and visibility of the loudspeakers: when (loud)speakers are visible, visual cues might dominate usage of ILDs and ITDs. An example is the strong ventriloquism effect (Hendrickx et al. 2015). Because of the dominance of visual cues in some listening situations, processing of ITDs and ILDs is typically investigated in complete darkness. However, assessment of sound localization abilities in a clinical setting is often performed using setups with visible loudspeakers.

Regarding the response method: it is well known that methodological differences in target pointing can affect the accuracy of a subject’s response (Bahu et al. 2016). The present data (Fig. 7) suggest that an indirect pointing method with an indicator box (clinic C) results in less accurate responses compared to simply head-oriented responses (all other clinics).

Regarding the cognitive feedback: participants were told that they performed poorly in condition 2 and that they localized most of the sounds toward the unplugged ear, while stimuli were presented from all speaker locations. This information by itself, without any additional information regarding the change in timbre, could have affected performance. Therefore, the nature of the manipulation requires further research.

## Conclusion

We demonstrate that localization abilities of monauralized normal-hearing listeners improved immediately after providing explicit information about the acute monaural hearing condition, when BB sounds were presented at a single stimulus level. The ability to improve monaural localization was not affected by small differences between the different test sites with regard to sound-localization setups, demonstrating how robust and generalizable the observed effect is.

We conclude that providing top-down information regarding the acute monaural listening situation in combination with information regarding the change in timbre that can be perceived in the monaural condition, instantly improves the localization abilities when loudspeakers are visible during closed-set testing. The results have important clinical implications and should be considered when investigating sound localization abilities, and when providing training, after treatment of (unilaterally) hearing-impaired patients.

## Abbreviations

BB: Broadband
ILDs: Interaural level differences
ITDs: Interaural time differences
MAE: Mean absolute error
